# Supplementary material for: Successful Prenatal Treatment of Cardiac Rhabdomyoma in a Fetus with Tuberous Sclerosis
Source: Pediatr Rep. 2023 Mar 22;15(1):245–53. doi: 10.3390/pediatric15010020 (PMC10059978; doi:10.3390/pediatric15010020)
Supplement: Supplementary file 1 [file pediatrrep-15-00020-s001.zip › pediatrrep-2258780-supplementary.pdf]

Author	Indication for Treatment	Start of Treatment (Gestational Weeks)	Maximal Tumor Size	Sirolimus Dose (mg/d)	Sirolimus Blood Level (ng/ml)
Park et al. 2019 [12]	Pulmonary LAM in mother	23	Multiple tumors max. diam. 12.4 mm	12 (until delivery)	Mother p.p.: 25.0<br>Cord blood: 33.2
Barnes et al. 2018 [10]	Bilateral outflow obstruction<br>SVT<br>Impending hydrops fetalis	30	Multiple tumors 320/290 mm^2	Not specified	Target trough: 10 –15<br>Mother p.p.: 6.9<br>Cord blood: 11.3
Pluym et al. 2019 [11]	LV inflow obstruction<br>Mitral valve regurgitation<br>Pericardial effusion<br>Impending hydrops fetalis	28	Multiple tumors 45 x 35 mm	6–10 (Stop 1 week before delivery)	Maternal trough level: 11.6 – 18.6<br>Mother p.p.: 3.4<br>Cord blood: 3.3
Vachon-Marceau et al. 2019 [13]	Biventricular dysfunction<br>Tricuspid valve insufficiency<br>Pericardial effusion	31 + 4 days	Multiple tumors 47 x 39 mm	5–8	Target trough level: 10 – 15
Ebrahimi-Fakhari et al. 2021 [14]	LV outflow obstruction	35 + 2 days	Multiple tumors Size not specified	3	Maternal trough level: 6.1
Ebrahimi-Fakhari et al. 2021 [14]	Tumor encapsulating LV	33 + 1 day	Multiple tumors 39 x 34 x 28 mm	3	Maternal trough level: 3.7
Ebrahimi-Fakhari et al. 2021 [14]	LV outflow obstruction	34	Multiple tumors Size not specified	6	Maternal trough level: 10.85 +/- 1.26 SD
Dagge et al. 2022 [15]	RV outflow obstruction<br>Tricuspid valve insufficiency<br>SVT	26	Single tumor 180 mm^2	4–10	Maternal trough level: Target 10 – 15; max. 16.7
Will et al. (this study)	Tricuspid valve stenosis + insufficiency<br>RV decompensation<br>Pericardial effusion	27	Multiple tumors<br>21,5 x 39,7 mm (RA)<br>18,5 x 29,9 mm (RV)	4 (Stop 8 days before delivery)	Maternal trough level: 8.4 – 9.9<br>Mother p.p: 1.16<br>Cord blood: 1.59

Table S1. Cases reported on prenatal sirolimus treatment for fetal rhabdomyoma. LV, left ventricle; RA, right atrium; RV, right ventricle; LAM, lymphangioleiomyomatosis; max. diam.; maximal diameter; p.p., post partum; SVT, supraventricular tachycardia.
